# Comparative efficacy and safety of prophylactic norepinephrine and phenylephrine in spinal anesthesia for cesarean section: A systematic review and meta-analysis with trial sequential analysis

**DOI:** 10.3389/fphar.2022.1015325

**Published:** 2022-11-28

**Authors:** Peng Liu, Hong He, Shan-Shan Zhang, Yun Liang, Zi-Jun Gao, Hui Yuan, Bu-Huai Dong

**Affiliations:** ^1^ Department of Anesthesiology, Xi’an Honghui Hospital, Affiliated Hospital of Xi’an Jiaotong University, Shaanxi, China; ^2^ Xi’an Medical University, Shaanxi, China; ^3^ Department of Anesthesiology, Sichuan Academy of Medical Sciences and Sichuan Provincial People’s Hospital, Chengdu, China; ^4^ Department of Anesthesiology, The First Affiliated Hospital of Xi’an Jiaotong University, Shaanxi, China

**Keywords:** norepinephrine, phenylephrine, cesarean section, spinal anesthesia, meta-analysis

## Abstract

**Background:** Phenylephrine is the first-line drug used to maintain blood pressure in cesarean delivery. However, it poses a high risk of bradycardia and depression of cardiac activity in pregnant women. Consequently, norepinephrine has gained popularity over the recent years, as an alternative to Phenylephrine because it is thought that prophylactic use of vasopressors may reduce the incidence of hypotension after spinal anesthesia. This systematic review compared the efficacy of both treatments.

**Methods:** We searched the following databases; CNKI, PubMed, Embase, Web of science, clinicaltrials.gov, Medline and Cochrane Library, for randomized controlled trials comparing the prophylactic efficacy of norepinephrine and phenylephrine on elective cesarean delivery under spinal anesthesia. The search period was from inception to July 2022, and the primary outcome indicator was incidence of bradycardia. Statistical analysis was conducted on Rev manager 5.4, and the Grading of Recommendations, Assessment, Development, and Evaluation (GRADE) framework was used to evaluate the quality of evidence from each main finding.

**Results:** A total of 12 papers were included in the analysis. The incidence of bradycardia (RR = 0.37, 95% CI: 0.28 to 0.49, *p* < 0.00001) and reactive hypertension (RR = 0.58, 95% CI 0.40 to 0.83, *p* = 0.003) was significantly lower in the norepinephrine (NE) group compared with the phenylephrine (PE) category. In contrast, there were no statistical differences in the umbilical cord blood gas analysis pH values between the groups (arterial: MD = 0.00, 95% CI −0.00 to 0.01, *p* = 0.22, vein: MD = 0.01, 95% CI −0.00 to 0.02, *p* = 0.06). The incidence of hypotension, nausea, and vomiting did not differ significantly between the NE and PE groups (hypotension: 23% vs. 18%; nausea: 14% vs. 18%; vomiting: 5% vs. 7%, respectively).

**Conclusion:** Prophylactic use of norepinephrine is safe and effective in maintaining maternal hemodynamics without causing adverse events to either the pregnant woman or fetus.

**Systematic Review Registration:** website https://www.crd.york.ac.uk/prospero/, identifier CRD42022347095

## 1 Introduction

Spinal anesthesia is commonly used for elective cesarean delivery. However, studies have shown that the hemodynamic changes caused by spinal anesthesia can affect up to 90% of pregnant women, and may lead to a series of adverse effects such as nausea, vomiting, and dizziness, or even threaten fetal safety (Kulkarni et al., 2016; Fitzgerald et al., 2020). Phenylephrine has, for a long time, been used as a pure α-receptor agonist to prevent hypotension induced by spinal anesthesia, especially in cesarean sections (Cho et al., 2020). However, its use may be accompanied by cardiac depression and bradycardia, resulting in a corresponding decrease in cardiac output, which is extremely detrimental to pregnant women with comorbidities (Stewart et al., 2010; Xu et al., 2018). On the other hand, Norepinephrine not only has both *α* and *ß* receptor agonist effects, but also confers a positive chronotropic effect on the heart (Ngan Kee et al., 2015). It has therefore gained popularity over the recent years, as a plausible alternative to phenylephrine for obstetric anesthesia. It is against this background that the present study sought to compare the efficacy of prophylactic norepinephrine and phenylephrine after spinal anesthesia for elective cesarean delivery.

Several meta-analyses have been published on either the efficacy of multiple antihypertensive agents in spinal anesthesia (Ryu et al., 2019; Singh et al., 2020), comparison of phenylephrine and ephedrine (Veeser et al., 2012; Heesen et al., 2019), or analyse the treatment of hypotension induced by spinal anesthesia (Kumari et al., 2022). However, our study focused on prophylaxis and not only highlights a different time point in the use of these drugs but also reflects a distinct line of thought. Furthermore, there is an international consensus on the prophylactic use of *α* agonists in spinal anesthesia for cesarean delivery (Kinsella et al., 2018) to prevent adverse events such as hypotension. Therefore, this study provides strong evidence to support clinical decisions regarding patient care during cesarean delivery.

## 2 Methods

### 2.1 Literature search

This meta-analysis was conducted based on the preferred reporting items and meta-analysis statements for systematic reviews (PRISMA) and the Cochrane Handbook for systematic reviews on interventions (Moher et al., 2009). Ethical approval or patient consent were not required because all analyses were based on previously published studies. The review was registered with PROSPERO (CRD42022347095).

A systematic search was conducted in the following databases; CNKI, PubMed, Embase, Web of science, clinicaltrials.gov, Medline, and Cochrane Library, using the following keywords: cesarean section (title/abstract) and spinal anesthesia (title/abstract) OR neuraxial anesthesia (title/abstract) OR lumbar anesthesia (title/abstract) OR subarachnoid block (title/abstract) OR intralesional anesthesia (title/abstract) AND norepinephrine OR phenylephrine (title/abstract) AND randomized controlled trial (RCT). The reference sections of respective articles were also explored for relevant literature to expand the search.

### 2.2 Study selection

The inclusion criteria were as follows: Cesarean delivery under elective intravesical anesthesia; the intervention involved prophylactic use of norepinephrine and phenylephrine, the article was peer-reviewed, and the study was a randomized controlled trial. The exclusion criteria were as follows: Emergency surgery, cesarean section performed under general anesthesia, interventions involving therapeutic use of norepinephrine and phenylephrine, non-RCT, and literature for which information on inclusion to the study could not be obtained and was inaccessible even after contacting the original authors.

### 2.3 Outcomes

The primary outcome indicator was the incidence of bradycardia (Heart rate<60 bpm). The secondary outcome indicators included: incidence of hypotension (systolic blood pressure (SBP) was <80% of the baseline or less than 90 mmHg), incidence of reactive hypertension (systolic blood pressure >120% of the baseline or SBP >140 mmHg), umbilical arterial blood gas pH, umbilical vein blood gas pH, and incidence of nausea and vomiting.

### 2.4 Data extraction

Two reviewers (LP and Z-SS) collated the final list of included studies and used a standardized data extraction format to obtain the data. After extraction, two other reviewers (YH and HH) matched the data, before re-reading the papers whenever discrepancies arose. Discrepancies were resolved through discussion with a third reviewer (D-BH or G-ZJ). The following information was included in the extracted data: first author, year, basic demographic characteristics, intervention protocol, and outcome indicators. If the required data were missing, not reported in the paper, or reported in an unusual form, the corresponding authors of the relevant papers were contacted for further clarification.

### 2.5 Quality assessment

We used the Cochrane risk of bias assessment tool to explore sources of bias in the included RCTs (Higgins et al., 2011). Using this tool, the risk of bias was evaluated during random sequence generation, allocation concealment, blinding of participants and researchers, blinding of the outcome assessments, selective reporting, incomplete outcome data, and other metrics. In addition, Funnel plot asymmetry tests, the Egger’s test, and the Begg-Mazumdar test were used to assess for potential evidence of reporting bias. Funnel plot asymmetry tests were only performed when there were at least ten studies (Sterne et al., 2011).

### 2.6 Evidence grade

The GRADE profiling system was employed to evaluate the quality of evidence for specific outcomes (Guyatt et al., 2008). The quality of evidence considers limitations, inconsistency, indirectness, imprecision, and risk of publication bias. Four levels of certainity are described in GRADE i.e., very low, low, moderate, and high.

### 2.7 Trial sequential analysis

Trial Sequential Analysis (TSA) is mainly used to assess the risk of Type I error in meta-analyses and whether there is a sufficient sample size to draw the current conclusions. We performed trial sequential analysis of the incidence of bradycardia (TSA Module version 0.9.5.10, Copenhagen trial unit, Denmark).

### 2.8 Methodological quality

We evaluated the methodological components of the included studies using a modified Jadad scale, where 1–3 was low quality while 4–7 was regarded as high quality. The evaluation included:1) Random sequence generation; 2) randomization concealment; 3) blinding; 4) withdrawal and exit. The first three items were judged as appropriate, unclear, and inappropriate, depending on the decision of the author.

### 2.9 Statistical analysis

The RevMan 5.4 software was used for statistical analysis. The risk ratio (RR) with 95% confidence intervals (CI) was calculated for dichotomous variables (binary outcomes) while the mean difference with 95% CI was estimated for continuous outcomes. If *p* ≥ 0.05 and I^2^ ≤ 50%, the difference in heterogeneity among studies was considered statistically insignificant, hence the meta-analysis was performed using a fixed-effects model. On the contrary, if *p* ≤ 0.05 and I^2^>50%, statistical heterogeneity among studies was considered, and the meta-analysis was performed using a random-effects model. The test level for the meta-analyses was set at *α* = 0.05. Moreover, the Egger’s and Begg’s tests were applied to quantitatively evaluate the significance of asymmetry. Notably, the umbilical cord blood gas analysis values from some studies were expressed as medians (quartiles) and could not be included in the meta-analysis. Therefore, the Box-Cox (BC) method was adopted to estimate the mean ± standard deviation as suggested by McGrath et al. (2020). Finally, sensitivity analysis was conducted to evaluate the stability of the results, by deleting each study individually.

## 3 Results

### 3.1 Literature search and the included studies

**FIGURE 1:**
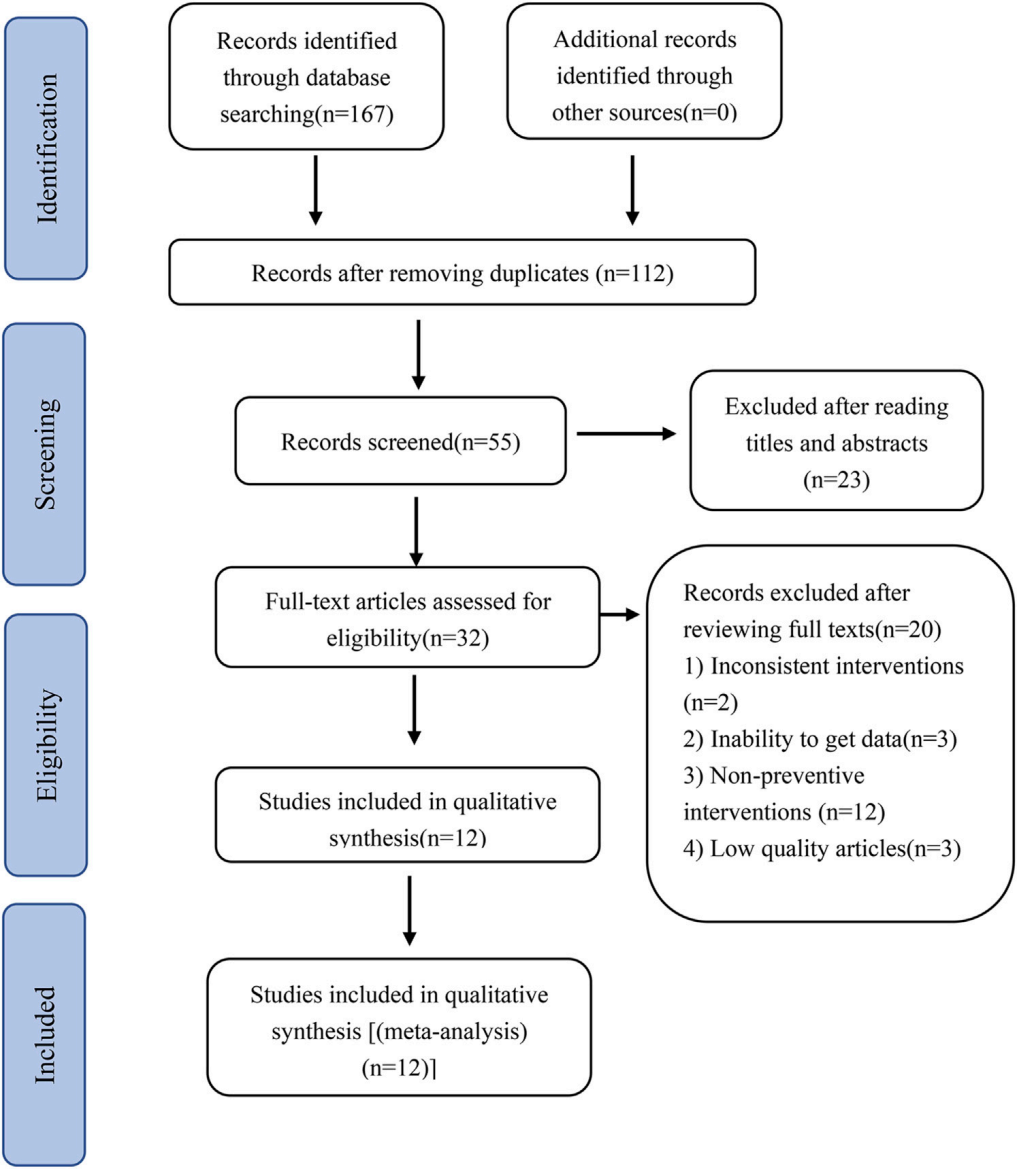
PRISMA flow chart of the article filtering process.

The PRISMA flow chart for the literature search is shown in Figure 1. A total of 167 articles were retrieved, out of which 112 duplicates were excluded, leaving 55 articles. After reading the titles and abstracts, 23 articles were excluded, and the remaining 32 were read in full. However, 20 articles were excluded further due to such reasons as low quality, and use of the drugs for therapeutic interventions, leaving 12 articles for inclusion in the study.

### 3.2 Study characteristics

Table 1 gives a summary of the characteristics of the included trials. A total of 12 studies (Dong et al., 2017; Vallejo et al., 2017; Hasanin et al., 2019; Sharkey et al., 2019; Theodoraki et al., 2020; Berawala et al., 2021; Eskandr et al., 2021; Chen et al., 2022; Du et al., 2022; Guo et al., 2022; Singh et al., 2022; Zhou et al., 2022) were included, all of which were published in the last 5 years. Five (Dong et al., 2017; Chen et al., 2022; Du et al., 2022; Guo et al., 2022; Zhou et al., 2022) of the studies were conducted in China, two (Berawala et al., 2021; Singh et al., 2022) in India, two (Hasanin et al., 2019; Eskandr et al., 2021) in Egypt, and the remaining three (Vallejo et al., 2017; Sharkey et al., 2019; Theodoraki et al., 2020) in Canada, the United States, and Greece, respectively, with participants ranging from 18 to 45 years. One of the studies involved an intervention in which a dose of the study drug was administered intravenously, immediately after spinal anesthesia, while the remaining administered the study drug at a fixed rate. The methodological component of the included studies was also assessed using a modified Jadad scale, and although only one study had a low score, it was still in the high-quality range. The remaining studies had high scores (Table 1).

**TABLE 1:** Characteristics of the included studies.

Study	Region	Age mean (SD)		Weight (kg)		Sample size		Interventions		Jadad score
		NE	PE	NE	PE	NE	PE	NE	PE	
Berawala et al. (2021)	India	24.31 (2.93)	25.88 (3.46)	63.42 (9.52)	59.34 (7 .27)	35	35	PNE	PPE	7
Eskandr et al. (2021)	Egypt	27.40 (3.71)	27.6 (4.00)	77.36 (6.92)	77.16 (6.39)	25	25	PNE	PPE	6
Hasanin et al. (2019)	Egypt	29 (25.33)	28 (24.31)	76 (61.90)	77 (60.91)	60	63	PNE	PPE	7
Guo et al. (2022)	China	18–45	18–45	NR	NR	69	69	PNE	PPE	4
Dong et al. (2017)	China	31.5 (3.5)	30.5 (4.9)	72.6 (8.1)	75.3 (8.3)	62	64	INE	IPE	7
Sharkey et al. (2019)	Canada	34.9 (4.7)	35.3 (3.9)	78.2 (11.7)	79.3 (10.6)	56	56	PNE	PPE	7
Singh et al. (2022)	India	27.57 (4.51)	26.20 (4.08)	59.72 (5.60)	60.96 (12.12)	30	30	PNE	PPE	7
Theodoraki et al. (2020)	Greece	30.6 (7.0)	33.2 (5.1)	77.4 (11.2)	77.2 (11.8)	41	41	PNE	PPE	7
Vallejo et al. (2017)	United States	30.2 (6.8)	29.1 (5.6)	NR	NR	43	38	PNE	PPE	7
Du et al. (2022)	China	32 (29–43.5)	32 (29.5–35)	75.48 (9.97)	73.62 (7.23)	31	31	PNE	PPE	7
Zhou et al. (2022)	China	32 (4)	32 (5)	NR	NR	25	25	PNE	PPE	7
Chen et al. (2022)	China	31.6 (4.3)	31.5 (4.2)	72.6 (8.0)	71.0 (11.2)	50	50	PNE	PPE	7

PNE: Immediately after spinal anesthesia, norepinephrine (NE) is pumped at a fixed rate; PPE: Immediately after spinal anesthesia, Phenylephrine (PE) is pumped at a fixed rate; INE: 10 µg of intravenous norepinephrine immediately after spinal anesthesia; IPE: 50 µg of intravenous norepinephrine immediately after spinal anesthesia. Age is expressed as Mean (SD) or median (quartiles); NR, not reported.

### 3.3 Risk of bias

**FIGURE 2:**
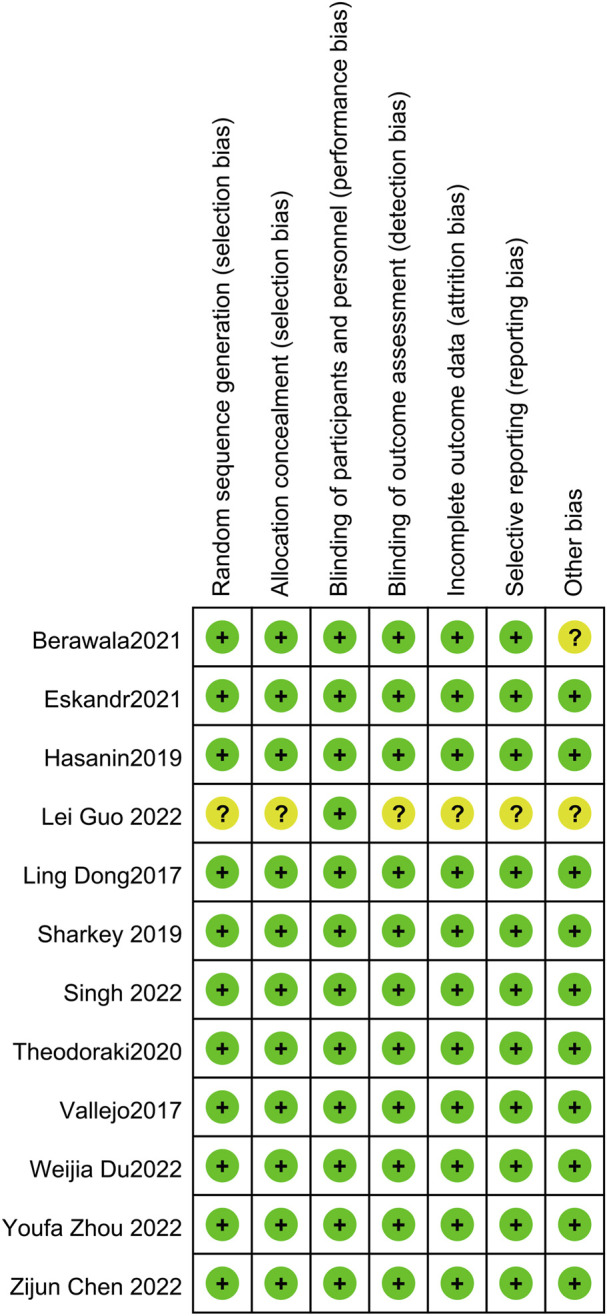
Risk of bias summary.

The risk of bias graphs for the included studies are shown in Figure 2. There was only one study (Guo et al., 2022) where the individual entries were not specified, hence judged as “unclear”, while in the rest of the studies, the entries were “low risk”.

### 3.4 Outcomes

#### 3.4.1 The incidence of bradycardia

**FIGURE 3:**
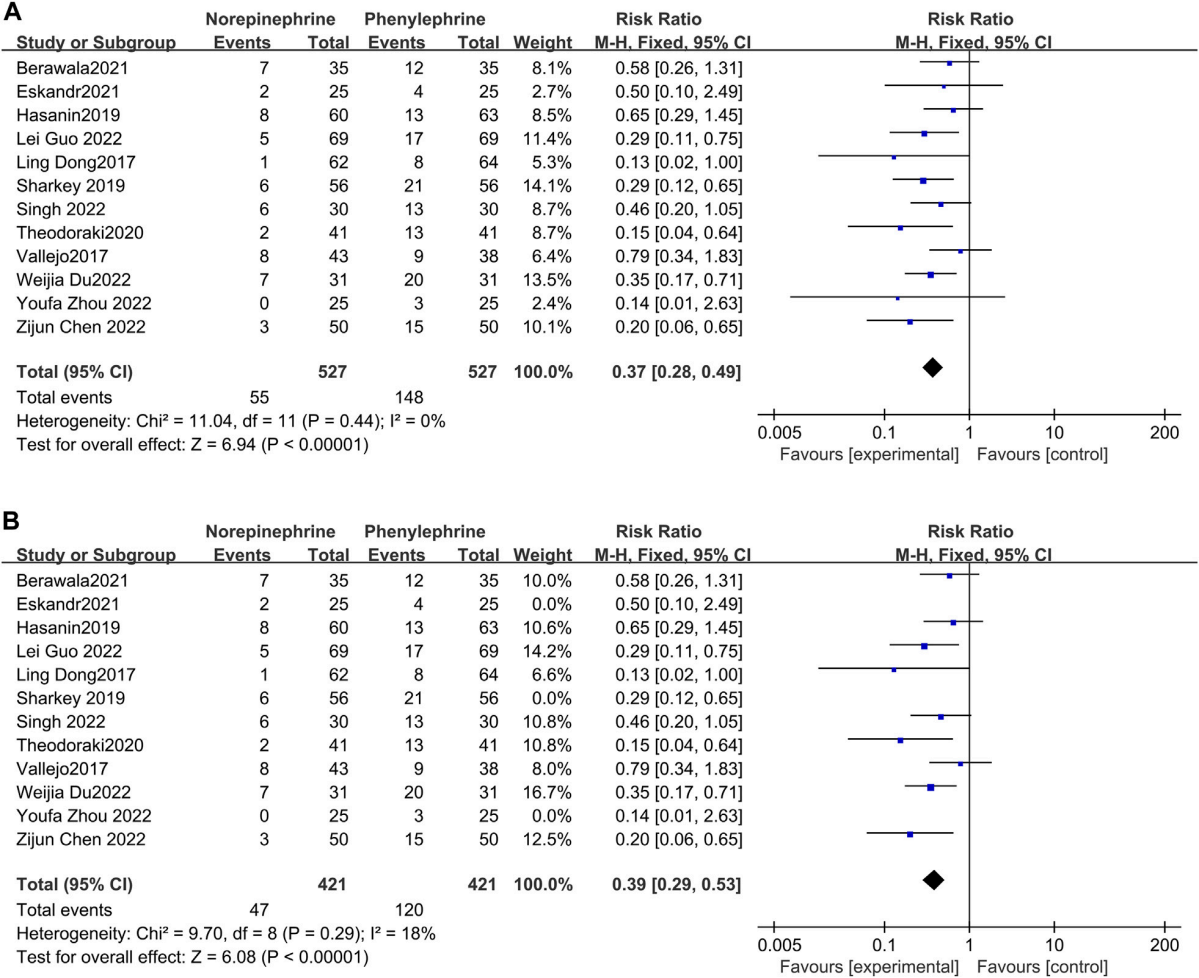
Forest plot of the incidence of bradycardia comparing norepinephrine with phenylephrine. **(A)** Analysis before exclusion. **(B)** Analysis after exclusion.

Pooled analysis of all the included studies showed that the incidence of bradycardia was recorded in all the 12 articles (Dong et al., 2017; Vallejo et al., 2017; Hasanin et al., 2019; Sharkey et al., 2019; Theodoraki et al., 2020; Berawala et al., 2021; Eskandr et al., 2021; Chen et al., 2022; Du et al., 2022; Guo et al., 2022; Singh et al., 2022; Zhou et al., 2022), with no significant heterogeneity between studies (*p* = 0.44, I^2^ = 0%). Meta-analysis using a fixed effects model showed that the incidence of bradycardia was significantly lower and statistically different in the prophylactic norepinephrine group compared to the phenylephrine category (RR = 0.37, 95% CI: 0.28 to 0.49, *p* < 0.00001), Figure 3 A. Bradycardia was defined as having less than 50 beats/min in three studies (Sharkey et al., 2019; Eskandr et al., 2021; Zhou et al., 2022), which were excluded from the analyses. The findings revealed no significant heterogeneity between the two groups (*p* = 0.29, I^2^ = 18%). Moreover, pooled data using a fixed effects model showed that the norepinephrine group had a significantly reduced incidence of bradycardia compared to the controls (RR = 0.39, 95% CI: 0.29 to 0.53, *p* < 0.00001), as shown in Figure 3B.

#### 3.4.2 The incidence of hypotension

**FIGURE 4:**
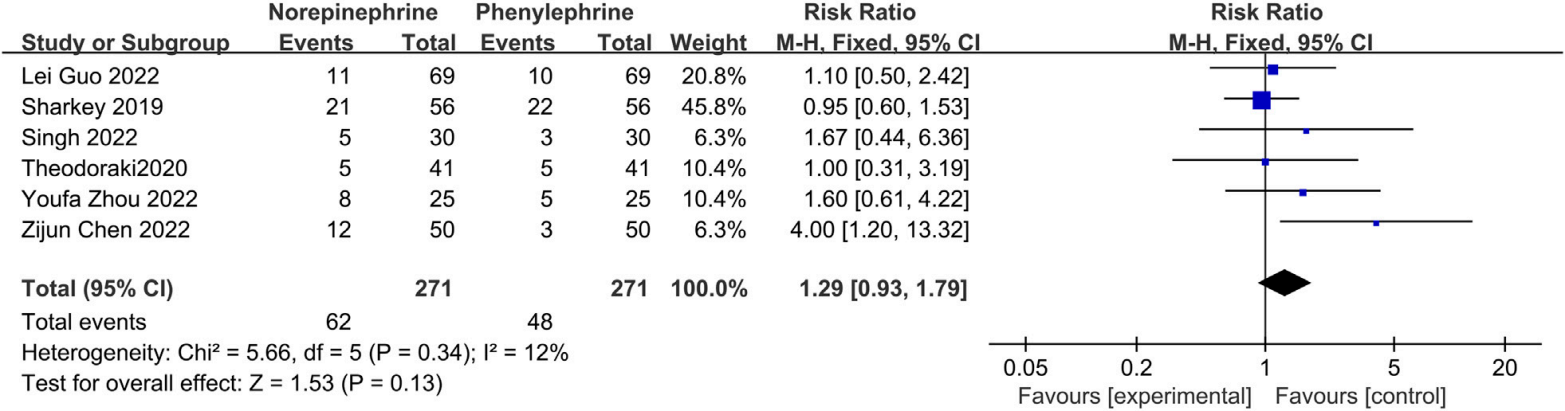
A Forest plot of the incidence of hypotension following treatment with either norepinephrine or phenylephrine.

In this analysis, data related to the incidence of hypotension was recorded in only six studies (Sharkey et al., 2019; Theodoraki et al., 2020; Chen et al., 2022; Guo et al., 2022; Singh et al., 2022; Zhou et al., 2022), with little heterogeneity between them (*p* = 0.34, I^2^ = 12%). Additionally, analysis using a fixed effects model showed that the incidence of hypotension between the experimental and control groups was comparable and not statistically different (RR = 1.29, 95% CI 0.93–1.79), Figure 4.

#### 3.4.3 The incidence of reactive hypertension

**FIGURE 5:**
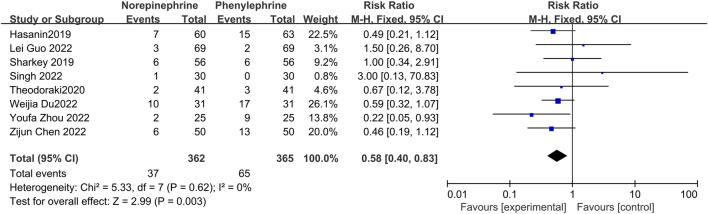
Forest plot of the incidence of reactive hypertension comparing norepinephrine with phenylephrine.

Reactive hypertension may occur after prophylactic use of vasopressors, and in this analysis, eight studies (Hasanin et al., 2019; Sharkey et al., 2019; Theodoraki et al., 2020; Chen et al., 2022; Du et al., 2022; Guo et al., 2022; Singh et al., 2022; Zhou et al., 2022) documented the occurrence of hypertension in both groups, with no significant heterogeneity between the included reports (*p* = 0.62, I^2^ = 0%). Analysis using a fixed effects model showed that the incidence of hypertension was significantly lower in the norepinephrine group than in the control category (RR = 0.58, 95% CI 0.40 to 0.83, *p* = 0.003), as shown in Figure 5.

#### 3.4.4 The incidence of nausea

**FIGURE 6:**
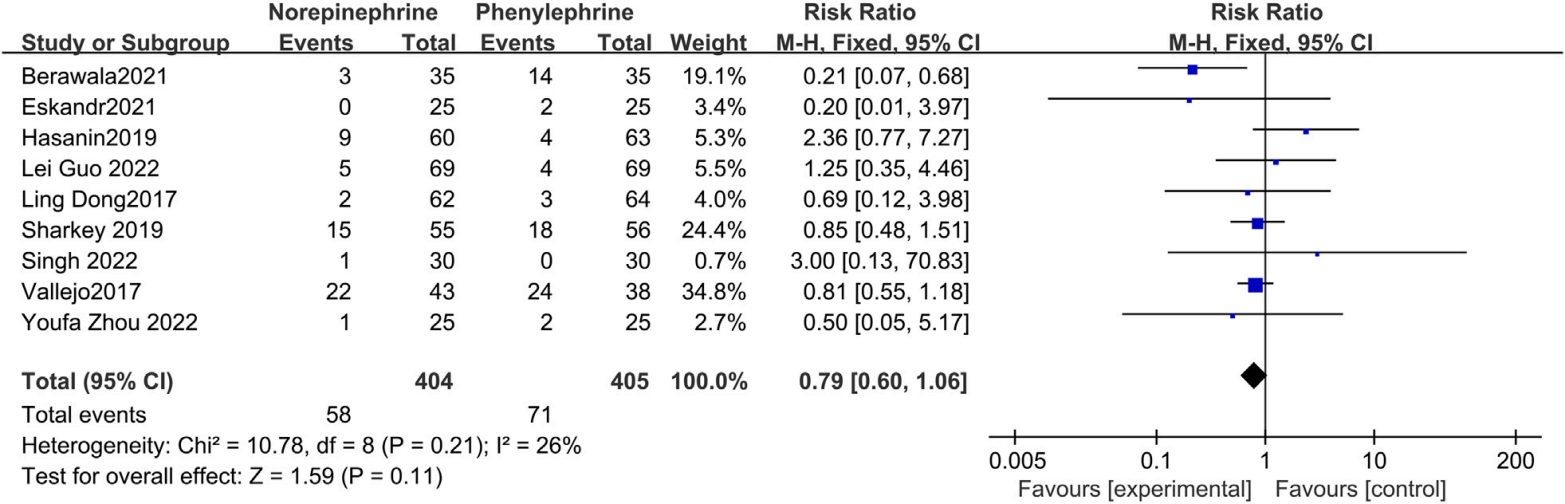
Forest plot of the incidence of nausea comparing norepinephrine with phenylephrine.

Data related to the incidence of nausea were pooled and recorded in nine studies (Dong et al., 2017; Vallejo et al., 2017; Hasanin et al., 2019; Sharkey et al., 2019; Berawala et al., 2021; Eskandr et al., 2021; Guo et al., 2022; Singh et al., 2022; Zhou et al., 2022) with minimal heterogeneity between the included articles (*p* = 0.21, I^2^ = 26%). The fixed effects model (Figure 6) showed no statistical difference in the incidence of nausea between the two groups (RR = 0.79, 95% CI 0.60 to 1.06, *p* = 0.11).

#### 3.4.5 The incidence of vomiting

**FIGURE 7:**
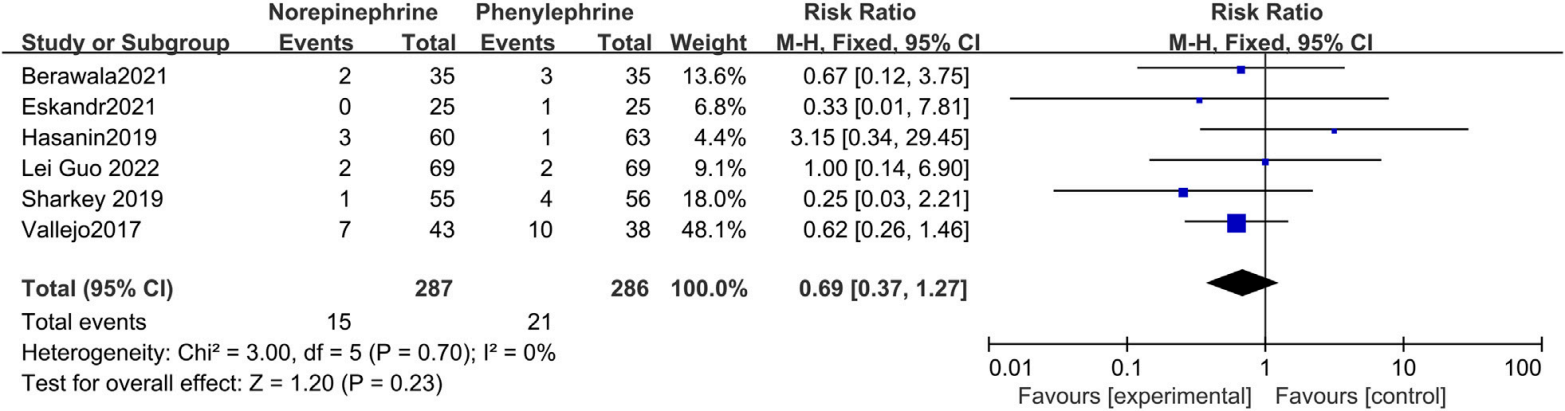
Forest plot of the incidence of vomiting comparing norepinephrine with phenylephrine.

Six studies reported on the occurrence of vomiting (Vallejo et al., 2017; Hasanin et al., 2019; Sharkey et al., 2019; Berawala et al., 2021; Eskandr et al., 2021; Guo et al., 2022) with no significant heterogeneity between them (*p* = 0.70, I^2^ = 0%). In addition, analysis using a fixed effects model showed that the occurrence of vomiting was not statistically different between the two groups (RR = 0.69, 95% CI 0.37 to 1.27, *p* = 0.23) (Figure 7).

#### 3.4.6 Umbilical arterial and vein blood gas analyses

**FIGURE 8:**
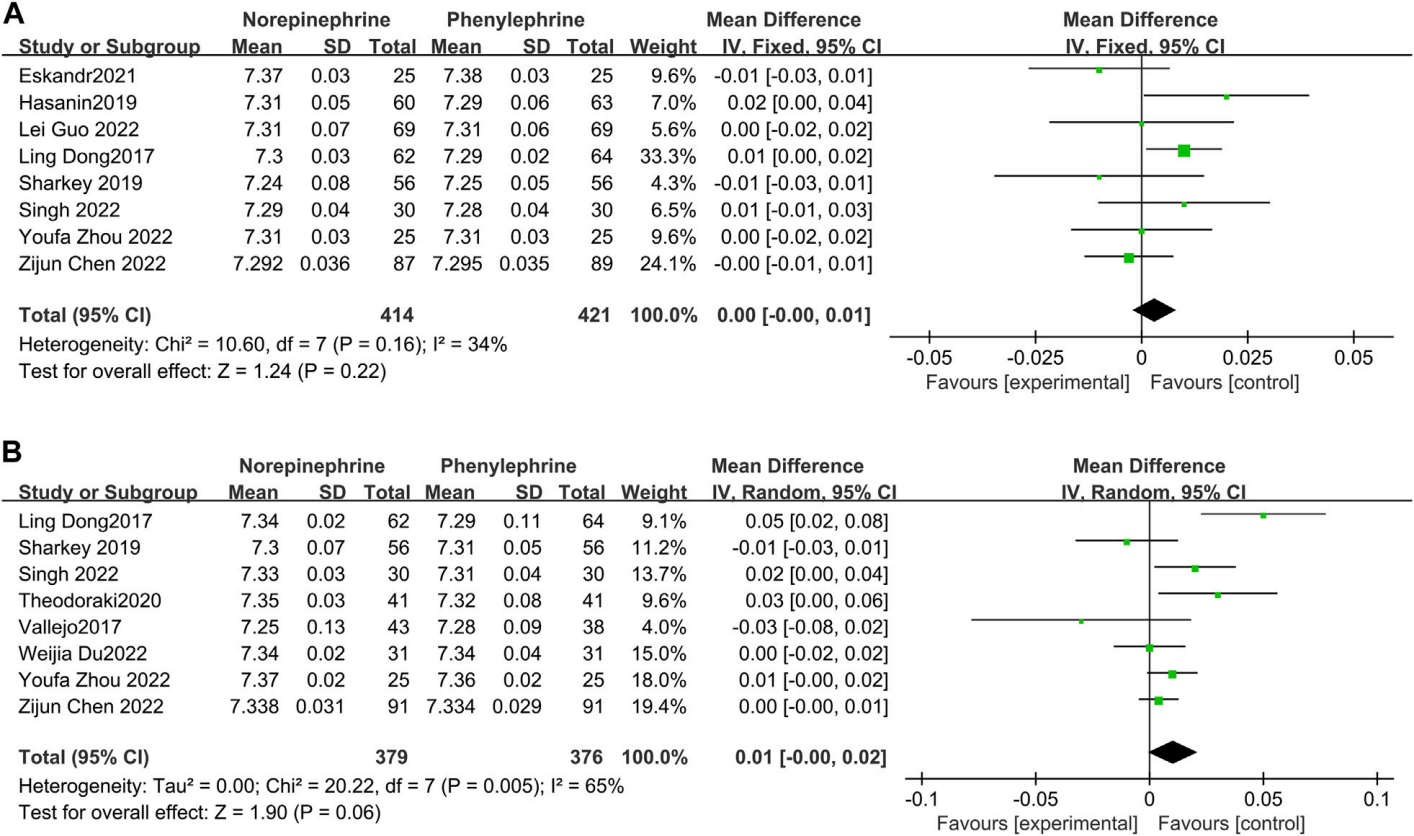
**(A)**: Umbilical arterial blood gas analyses; **(B)**: Umbilical vein blood gas analyses.

The blood gas analysis pH values of the included studies was extracted and 8 (Dong et al., 2017; Hasanin et al., 2019; Sharkey et al., 2019; Eskandr et al., 2021; Chen et al., 2022; Guo et al., 2022; Singh et al., 2022; Zhou et al., 2022) recorded the umbilical artery blood gas pH. Further analysis revealed minimal heterogeneity between the included studies (*p* = 0.16, I^2^ = 34%), and the fixed effects model showed that there was no statistical difference in umbilical artery blood gas pH values between the two groups (MD = 0.00, 95% CI −0.00 to 0.01,*p* = 0.22). Eight studies (Dong et al., 2017; Vallejo et al., 2017; Sharkey et al., 2019; Theodoraki et al., 2020; Chen et al., 2022; Du et al., 2022; Singh et al., 2022; Zhou et al., 2022) recorded the umbilical vein blood gas pH values, with great heterogeneity among the included reports (*p* = 0.005, I^2^ = 65%). Moreover, the random effects model showed that there was no significant difference in umbilical vein blood gas pH values between the two groups (MD = 0.01, 95% CI −0.00 to 0.02, *p* = 0.06), Figure 8.

### 3.5 Sensitivity analysis

There was great heterogeneity in the cord blood gas pH values among the included studies. Sensitivity analysis was then conducted by excluding the studies one by one, and there was still great heterogeneity among the reports. The source of heterogeneity was further investigated and it was discovered that some studies reported the pH values as medians (quartiles) (Dong et al., 2017; Vallejo et al., 2017; Hasanin et al., 2019; Theodoraki et al., 2020; Du et al., 2022) yet the present analysis used a Box-Cox (BC) formula for estimating the mean ± standard deviation, followed by meta-analysis. This difference in the estimation method was thought to be possible source of heterogeneity.

### 3.6 Publication bias detection

**FIGURE 9:**
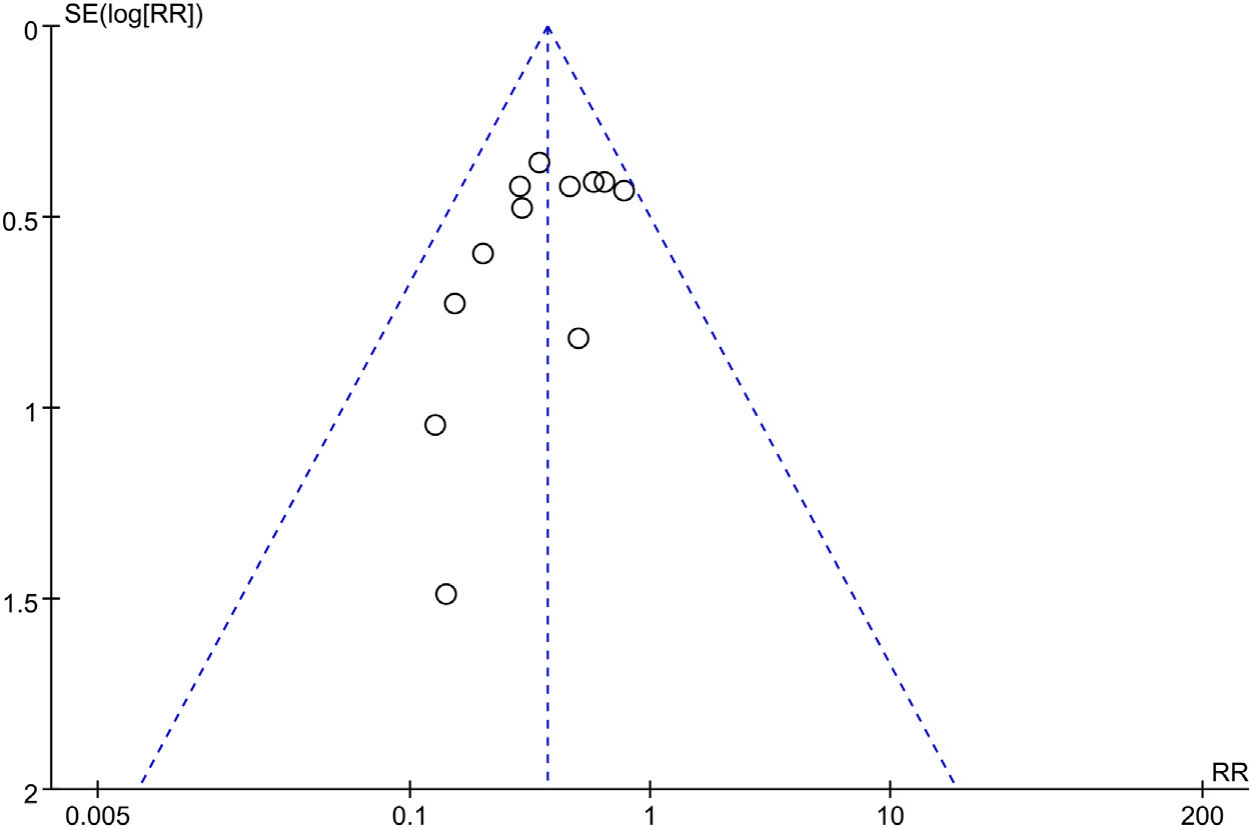
Funnel plot of the incidence of bradycardia.

The Egger’s and Begg’s tests in Stata 16.0 were used to evaluate publication bias for the incidence of bradycardia. Both tests [Egger’s test (*p* = 0.1618) and Begg’s test (*p* = 1.8074)] revealed that there was no publication bias. We also generated a funnel plot (Figure 9), which was not uniformly distributed, highlighting potential bias.

### 3.7 Trial sequential analysis

**FIGURE 10:**
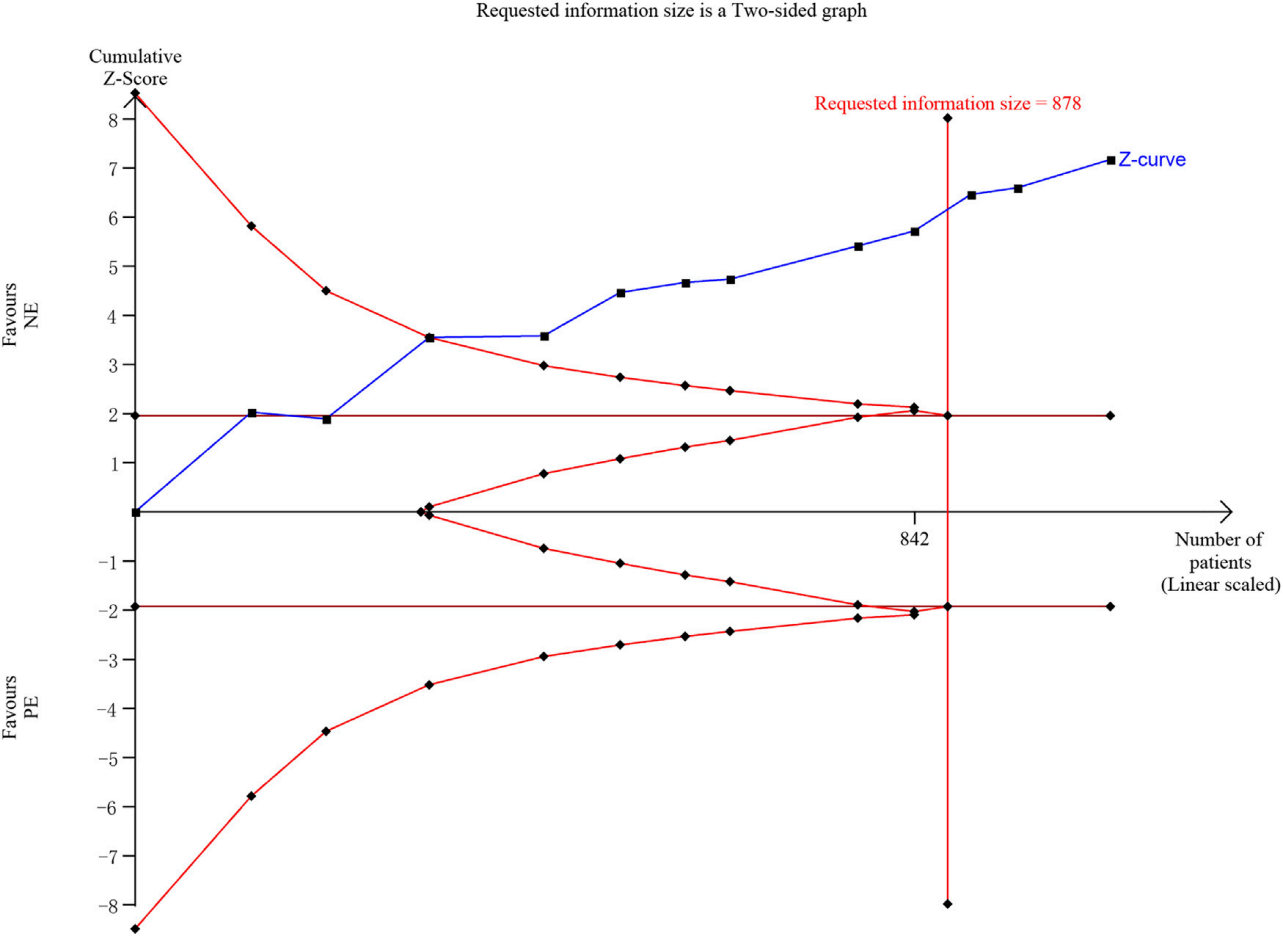
Trial sequential analysis of the incidence of bradycardia. We calculated a spending-adjusted required information size (RIS) using a = 0.05 (two-sided), and power = 80%. Blue—the cumulative Z-curve; Deep red—the conventional boundary; red—the TSA boundary.

We performed a TSA of the incidence of bradycardia, and the analysis showed that the cumulative Z-curve had crossed the TSA boundary as well as the RIS (required information size), confirming the ability of NE to reduce the incidence of bradycardia (Figure 10).

### 3.8 GRADE assessment

Some of the studies (Guo et al., 2022) had no details on randomization, and there was some degree of heterogeneity in both the incidence of nausea and umbilical artery blood gas pH, although within acceptable limits. There was however greater heterogeneity in umbilical vein blood gas pH values, exceeding 50%, so we reduced the quality of the associated evidence in accordance with the GRADE recommendations (Table 2).

**TABLE 2:** Levels of evidence for outcome indicators.

Outcome	Limitations	Inconsistency	Indirectness	Imprecision	Publication bias	Conclusion	Quality of evidence (GRADE)
The incidence of bradycardia	Serious limitations^a^	No serious inconsistency	No serious indirectness	No serious imprecision	No publication bias	Reduced the incidence of bradycardia	Moderate quality
The incidence of hypotension	Serious limitations^a^	No serious inconsistency	No serious indirectness	No serious imprecision	No publication bias	No significant difference	Moderate quality
The incidence of reactive hypertension	Serious limitations^a^	No serious inconsistency	No serious indirectness	No serious imprecision	No publication bias	Reduced the incidence of reactive hypertension	Moderate quality
The incidence of nausea	Serious limitations^a^	No major inconsistency^b^	No serious indirectness	No serious imprecision	No publication bias	No significant difference	Moderate quality
The incidence of vomiting	serious limitations^a^	No serious inconsistency	No serious indirectness	No serious imprecision	No publication bias	No significant difference	Moderate quality
Umbilical arterial blood gas analyses	Serious limitations^a^	No major inconsistency^b^	No serious indirectness	No serious imprecision	No publication bias	No significant difference	Moderate quality
Umbilical vein blood gas analyses	Serious limitations^a^	Serious inconsistency^c^	No serious indirectness	No serious imprecision	No publication bias	No significant difference	Low quality

^a^ Some studies had no details on randomization.

^b^ Some studies had some heterogeneity, but within acceptable limits.

^c^ Some studies I^2^ > 50%.

## 4 Discussion

This study found that prophylactic use of norepinephrine significantly reduces the incidence of bradycardia and reactive hypertension compared to treatment with phenylephrine. However, the incidence of hypotension, nausea and vomiting was similar between the two treatments (hypotension: 23% vs. 18%; nausea: 14% vs. 18%; vomiting: 5% vs. 7%, respectively). These findings provide evidence supporting the use of norepinephrine as an alternative to phenylephrine.

Advancement in medicine has enabled a better understanding of the mechanism underlying the occurrence of hypotension caused by spinal anesthesia. This has led to improvements in prevention and treatment strategies. After spinal anesthesia, sympathetic nerves are blocked, microscopic arteries are dilated, and blood is pooled in the lower extremities, causing a decrease in cardiac output. These events eventually lead to hypotension, with an incidence of up to 75% (Wang et al., 2018; Sklebar et al., 2019). As such, it is reasonable to consider the contractile properties of vasopressors. According to existing reports, the incidence of hypotension is so common that routine prophylactic use is recommended (Kinsella et al., 2018). The present study chose bradycardia as the primary indicator because it is more common in clinical practice. Our findings suggest that prophylactic use of norepinephrine results in a lower incidence of bradycardia and reactive hypertension. Reactive hypertension may be a problem and is associated with prophylactic infusions of vasopressors, which in theory should be avoided, but in practice cannot be predicted. Moreover, some studies have shown that even single injections of drugs can cause reactive hypertension in a dose-dependent fashion (Allen et al., 2010). Consistent with findings from numerous previous studies, this meta-analysis showed that prophylactic use of norepinephrine is beneficial in maintaining maternal and fetal hemodynamic stability, reducing the incidence of adverse events, and providing stronger protection for fetal delivery.

The high incidence of nausea and vomiting during spinal anesthesia is mainly due to acute hypotension, which decreases cerebral perfusion, induces temporary brainstem ischemia and activates the vomiting center (Borgeat et al., 2003), potentially leading to transient cerebral hypoxia. Existing studies show that among the previously used vasopressors, phenylephrine is associated with a lower incidence of nausea and vomiting when used in cesarean delivery. Our study mainly compared the incidence of nausea and vomiting in the experimental and control groups, and showed no statistical difference between the two, suggesting that norepinephrine does not increase the associated adverse events. The analysis also compared the cord blood gas pH values, since some vasopressors have been reported to affect the fetal acid-base environment, e.g., ephedrine which may potentially cause fetal acidosis (Massoth et al., 2020). Additionally, a network meta-analysis confirmed that use of phenylephrine in cesarean delivery poses a lower risk of fetal acidosis (Singh et al., 2020). Similarly, our analysis confirmed that norepinephrine does not increase the risk of fetal acidosis, based on the cord blood gas PH values. In addition, existing literature indicates that norepinephrine indeed causes no change in the incidence of fetal acidosis (Massoth et al., 2020), further confirming its safety and effectiveness for use in cesarean delivery. It is recommended that an ideal vasopressor and its regimen should minimize maternal symptoms such as nausea, vomiting and fetal acidosis. Our analysis confirms that norepinephrine fits this recommendation, hence suitable for preventing hypotension in cesarean delivery.

While similar meta-analyses exist, they are not entirely identical to the present study. For instance, several meta-analyses have been published on either the efficacy of multiple antihypertensive agents in spinal anesthesia (Ryu et al., 2019; Singh et al., 2020), on the comparison of phenylephrine and ephedrine (Veeser et al., 2012; Heesen et al., 2019), or on the treatment of hypotension induced by spinal anesthesia a (Kumari et al., 2022). In contrast, the present study mainly focused on the use of norepinephrine prophylactically, immediately after spinal anesthesia and directly compared the efficacy of the two drugs. This therefore provides strong evidence to support the choice of norepinephrine in caesarian delivery, a finding that is not reported in existing meta-analyses.

Despite the insightful findings, this study had some drawbacks. First, the existing literature is limited and number of patients fairly low, highlighting the need for more clinical trials to confirm these findings. Second, the outcome indicators largely focused on mothers, and those related to newborns were not analyzed. Such include the apgar scores, which were all expressed as medians (quartiles) and could not be estimated. However, all the included studies revealed no significant differences between the apgar scores at 1 min and 5 min. We intended to conduct further confirmatory analyses in future studies. Third, there was some variation in the criteria for judging bradycardia, leading to potential bias, and the results should therefore be interpreted with caution. Fourth, some literature suggests that continuous infusion of norepinephrine affects fetal lactate levels, which may also cause some degree of potential difference. Finally, the analysis did not identify the optimal dose and mode of administration for norepinephrine for prophylactic use because of inconsistencies in the doses used across studies. Nonetheless, a previous study suggests that continuous intravenous infusion with fluids may be the optimal mode of demonstration (Allen et al., 2010).

In conclusion, the prophylactic use of norepinephrine during spinal anesthesia can be safely and effectively applied to pregnant women, significantly reducing hemodynamic fluctuations without increasing the risk of adverse events in both the mother and fetus.

## Data Availability

The original contributions presented in the study are included in the article/Supplementary Material, further inquiries can be directed to the corresponding author.
